# Rapidly Progressive Impaired Consciousness in Cervical Intramedullary Spinal Cord Abscess: A Case Report

**DOI:** 10.31662/jmaj.2024-0385

**Published:** 2025-06-06

**Authors:** Yuma Hiratsuka, Yasufumi Ohtake, Hirohiko Nakamura

**Affiliations:** 1 Department of Neurosurgery, Nakamura Memorial Hospital, Sapporo, Japan

**Keywords:** intramedullary spinal cord abscess, spinal surgery, antibiotic treatment, case report

## Abstract

Intramedullary spinal cord abscesses rarely cause impaired consciousness without intracranial infection. We report the case of a 65-year-old woman who presented with neck pain and right upper limb weakness. She developed rapidly progressive impaired consciousness within three days of admission. Initial magnetic resonance imaging revealed a right-sided intramedullary lesion at C4-C5 with surrounding edema extending to the brainstem. Brain imaging showed no abnormalities. An initial diagnosis of a spinal cord tumor was made. Follow-up imaging demonstrated lesion expansion with ring enhancement. The patient subsequently developed impaired consciousness and quadriplegia. Emergency surgery revealed and drained a spinal cord abscess, with cultures growing α-hemolytic Streptococcus. The patient’s consciousness improved quickly after surgery. Previously undiagnosed type 2 diabetes was identified during admission. Despite extensive investigation, no obvious source of infection was found. Antibiotic therapy with ampicillin was continued for one month. Imaging at two months showed complete resolution of the abscess. The patient regained independent walking ability after rehabilitation, with only slight residual right arm weakness. No recurrence was observed during ten years of follow-up. This case demonstrates that a cervical spinal cord abscess can cause impaired consciousness through brainstem edema without intracranial infection. Early surgical intervention combined with appropriate antibiotics may lead to favorable outcomes.

## Introduction

Intramedullary spinal cord abscesses (ISCAs) represent a rare but significant infection of the central nervous system. The clinical presentation typically includes progressive neurological deficits, which may resemble neoplastic or immune-mediated disorders ^(1)^. Magnetic resonance imaging (MRI) with gadolinium enhancement serves as the primary diagnostic tool, and treatment typically includes antibiotics and surgery. Recent evidence suggests improved outcomes with surgical intervention within 24 hours of diagnosis ^(1)^. However, the optimal treatment approach remains unclear due to the limited number of reported cases. Some cases are complicated by impaired consciousness. According to previous reports, impaired consciousness is primarily caused by concurrent brain infection ^(2), (3)^. Here, we report a case of a cervical spinal cord abscess that presented with impaired consciousness, likely due to abscess expansion alone without apparent spread of infection to the brain.

## Case Report

**Figure 1.**
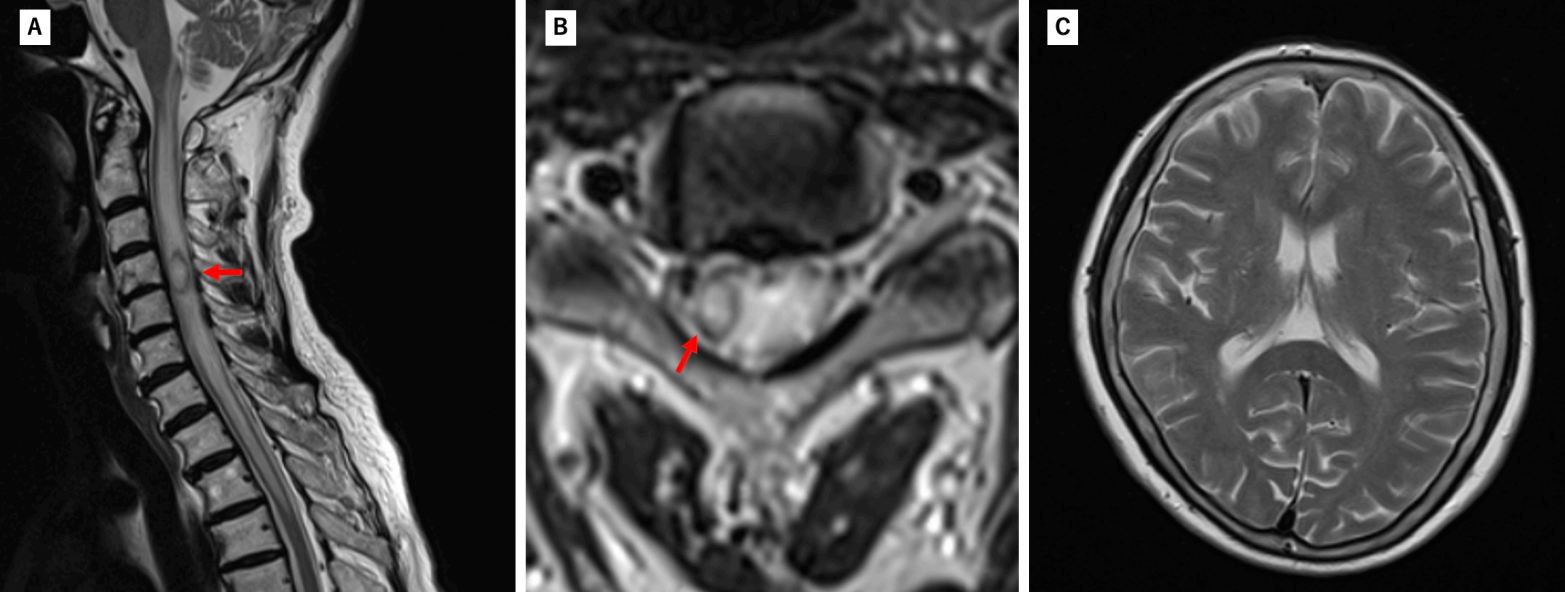
MRI on admission revealed a hyperintense intramedullary lesion (red arrow) on the right side at the C4-C5 spinal level with extensive perilesional edema extending to the brainstem. Brain MRI showed no obvious abnormalities. (A: T2WI Sagittal; B, C: T2WI Axial). MRI: magnetic resonance imaging; T2WI: T2 weighted image.

**Figure 2.**
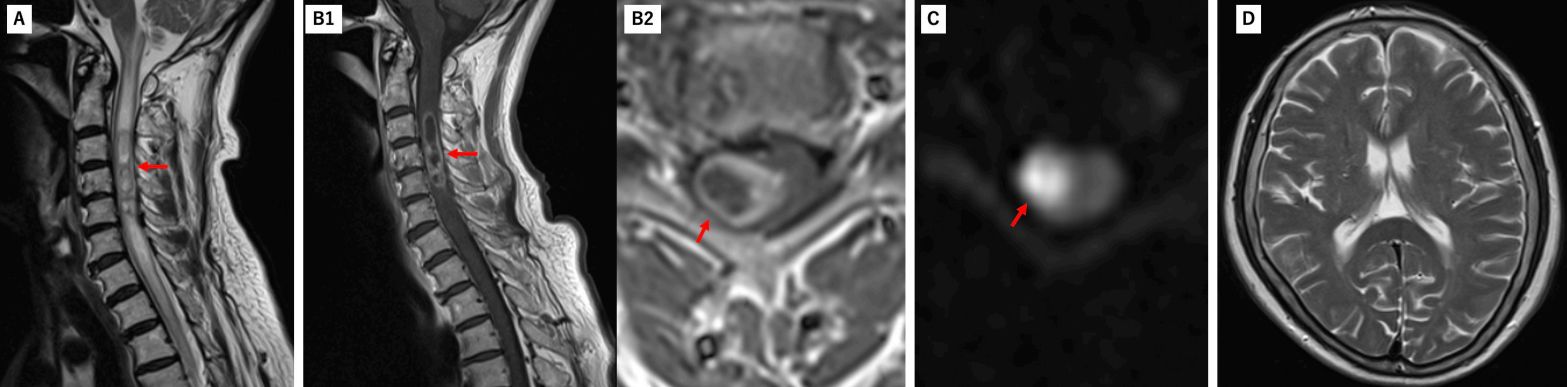
On the second day of hospitalization, a follow-up MRI showed progressive lesion expansion (red arrow) with worsening edema and ring-like enhancement on contrast-enhanced MRI, and high signal intensity on diffusion-weighted imaging in the same region. Brain MRI showed no changes compared to admission, with no hydrocephalus or findings suggestive of brain abscess or ventriculitis. (A: T2WI Sagittal; B1, 2: gadolinium-enhanced T1WI Sagittal, Axial; C: DWI Axial; D: T2WI Axial). MRI: magnetic resonance imaging; T2WI: T2 weighted image.

A 65-year-old woman presented with acute-onset neck and bilateral shoulder pain, accompanied by progressive right upper limb weakness over three days. She had a history of dyslipidemia and iron-deficiency anemia but no recent infections, trauma, surgeries, or immune system disorders. Initial examination revealed mild right upper limb weakness, normal vital signs, and no fever. Laboratory tests showed a white blood cell count of 6,900/μL and a C-reactive protein level below 0.05 mg/dL. MRI showed a right-sided intramedullary lesion at the C4-C5 level with surrounding edema extending to the brainstem, with no evidence of intracranial abnormalities (Figure 1). Initially diagnosed as a spinal cord tumor, the patient received corticosteroid therapy. However, follow-up MRI on the second hospital day demonstrated lesion expansion with increased edema on T2-weighted images and ring enhancement on contrast-enhanced T1-weighted images, while diffusion-weighted imaging showed high signal intensity in the same region. Brain MRI remained unremarkable (Figure 2). These findings prompted the initiation of broad-spectrum antibiotic therapy with meropenem. On the third hospital day, the patient deteriorated neurologically, presenting with altered consciousness (GCS9 E2V3M4) and quadriparesis, necessitating emergency surgical intervention. Cervical laminectomy revealed a spinal cord abscess, which was drained and sampled for pathological examination and culture. α-hemolytic Streptococcus was isolated from the surgical cultures, confirming the diagnosis. The patient’s consciousness improved rapidly after surgery, with oral intake resuming on postoperative day two and gradual recovery of neurological function. Despite an extensive workup, no other obvious source of infection was identified; however, type 2 diabetes was diagnosed during admission, requiring oral hypoglycemic treatment.

**Figure 3.**
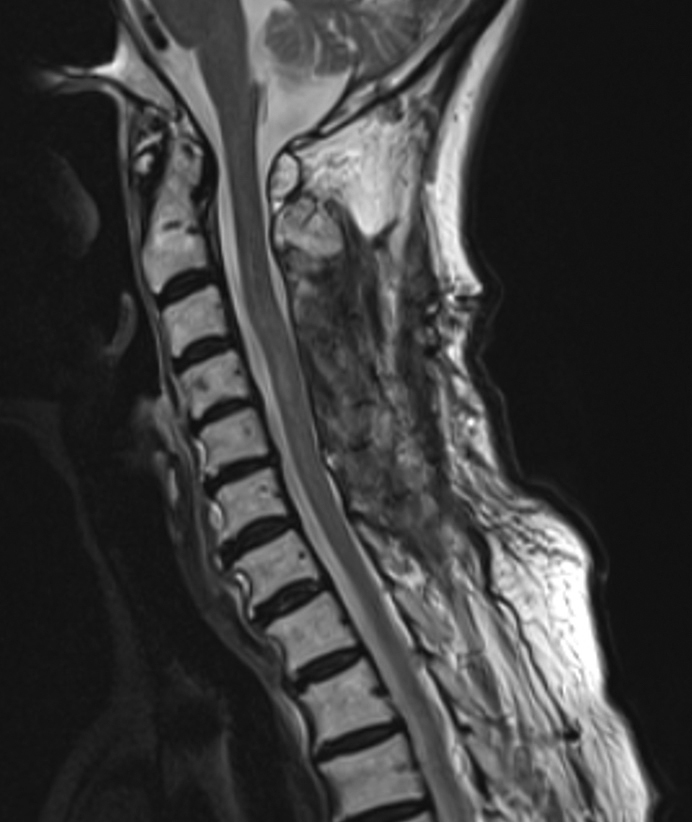
Two months after surgery, the MRI revealed complete resolution of the abscess with resolution of surrounding edema. (T2WI Sagittal). MRI: magnetic resonance imaging; T2WI: T2 weighted image.

The antibiotic was de-escalated to ampicillin and continued for one month. Two months after surgery, MRI revealed complete resolution of the abscess along with surrounding edema (Figure 3). Two months of intensive rehabilitation restored the patient’s ability to walk independently. At discharge, she maintained independence in daily activities, with only slight residual right arm weakness. A ten-year follow-up revealed no recurrence.

## Discussion

This case of ISCA illustrates several crucial aspects of managing this rare condition. The speed of neurological deterioration serves as a key diagnostic indicator for ISCAs ^(4)^, which can help distinguish them from slower-growing neoplasms ^(5)^, although initial diagnosis remains challenging due to their similarity with tumors.

**Figure 4.**
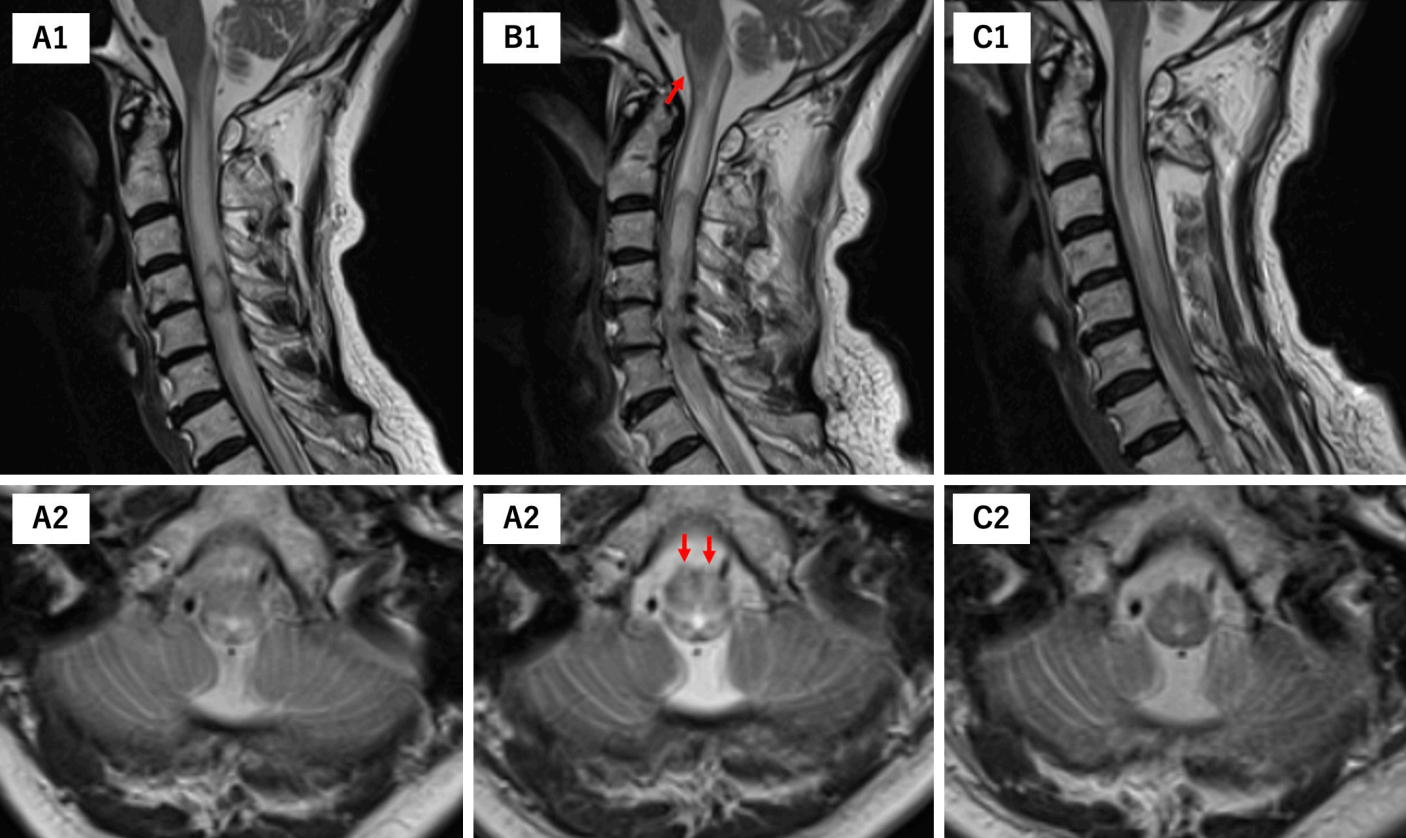
Initial MRI showed edematous changes limited to the dorsal brainstem (A1, 2). When the patient developed impaired consciousness, the MRI revealed that the edema had spread to the central and ventral medulla (red arrows) (B1, 2). Postoperative imaging showed improvement in brainstem edema (C1, 2), which was accompanied by recovery of consciousness. (A1, B1, C1: T2WI Sagittal; A2, B2, C2: T2WI Axial). MRI: magnetic resonance imaging; T2WI: T2 weighted image.

Various mechanisms of consciousness disturbance in cervical ISCA cases have been reported, including brain abscesses and ventriculitis ^(2), (3)^. Despite the absence of other intracranial abnormalities, edematous changes extended to the central and ventral medulla (Figure 4), possibly affecting the medullary reticular formation, which likely caused the patient’s impaired consciousness ^(6)^. This hypothesis was further supported by the rapid improvement in consciousness level following surgical drainage. This clinical course aligns with previous studies that identified prompt treatment, consisting of appropriate antimicrobial therapy with surgical drainage, as a crucial factor for improved outcomes ^(7)^, particularly in cases where consciousness disturbance is primarily caused by reversible edematous changes in the brainstem rather than direct infection of intracranial structures.

The patient was diagnosed with type 2 diabetes during hospitalization, which may have contributed to the development of ISCA, though the source of infection remained cryptogenic, this finding is consistent with previous reports, in which 64% of spinal cord abscesses had no identifiable source ^(8)^.

Furthermore, as previously reported in the literature, ISCA can present without typical signs of bacterial infection, such as fever or elevated inflammatory markers ^(8), (9)^. The MRI finding of ring enhancement should have prompted earlier consideration of immediate surgical drainage along with antibiotic therapy. This underscores the importance of maintaining a high index of suspicion for ISCA even in the absence of typical inflammatory signs, particularly when characteristic imaging findings are present.

### Conclusion

Cervical spinal cord abscess might cause impaired consciousness associated with brainstem edema. Early surgical drainage combined with antibiotics may facilitate rapid recovery, even in cases without typical inflammatory signs.

## Article Information

### Conflicts of Interest

None

### Author Contributions

Substantial contributions to the conception or design of the work; or the acquisition, analysis, or interpretation of data for the work: Yuma Hiratsuka. Drafting the work or reviewing it critically for important intellectual content: Yuma Hiratsuka, Yasufumi Ohtake, and Hirohiko Nakamura. Final approval of the version to be published: Yasufumi Ohtake, Hirohiko Nakamura. Agreement to be accountable for all aspects of the work in ensuring that questions related to the accuracy or integrity of any part of the work are appropriately investigated and resolved: Yuma Hiratsuka, Yasufumi Ohtake, Hirohiko Nakamura

### Approval by Institutional Review Board (IRB)

2024081703, Nakamura Memorial Hospital

### Informed Consent

The patient provided written informed consent for her case to be published in an academic journal while maintaining anonymity.

## References

[1] Harrold GK, Ali AS, Berkowitz AL, et al. Clinical features and diagnosis of intramedullary spinal cord abscess in adults: a systematic review. Neurology. 2023;101(8):e836-44. 37400243 10.1212/WNL.0000000000207515 PMC10449440

[2] Sinha P, Parekh T, Pal D. Intramedullary abscess of the upper cervical spinal cord. Unusual presentation and dilemmas of management: case report. Clin Neurol Neurosurg. 2013;115(9):1845-50. 23453154 10.1016/j.clineuro.2013.01.008

[3] Virtanen PS, Jimenez MJD, Horak VJ, et al. Concomitant brain abscess and spinal cord abscess in an immunocompetent teenage male: illustrative case. J Neurosurg Case Lessons. 2023;5(4):CASE22458. 36692066 10.3171/CASE22458 PMC10550703

[4] Ehara T, Suzuki T, Mizuno R, et al. Rapidly progressing intramedullary spinal cord abscess: a case report. NMC Case Rep J. 2024;11:43-7. 38454915 10.2176/jns-nmc.2023-0144 PMC10917654

[5] Ul Haq N, Hassan M, Ali Z, et al. Intramedullary thoracic spinal cord abscess mimicking an intramedullary tumor: a case report. Cureus. 2023;15(8):e43387. 37700988 10.7759/cureus.43387 PMC10495102

[6] Parvizi J, Damasio A. Consciousness and the brainstem. Cognition. 2001;79(1-2):135-60. 11164026 10.1016/s0010-0277(00)00127-x

[7] Jabbar R, Szmyd B, Jankowski J, et al. Intramedullary spinal cord abscess with concomitant spinal degenerative diseases: a case report and systematic literature review. J Clin Med. 2022;11(17):5148. 36079075 10.3390/jcm11175148 PMC9457049

[8] Hood B, Wolfe SQ, Trivedi RA, et al. Intramedullary abscess of the cervical spinal cord in an otherwise healthy man. World Neurosurg. 2011;76(3-4):361.e15-9. 10.1016/j.wneu.2010.01.013 21986440

[9] Cerecedo-Lopez CD, Bernstock JD, Dmytriw AA, et al. Spontaneous intramedullary abscesses caused by Streptococcus anginosus: two case reports and review of the literature. BMC Infect Dis. 2022;22(1):141. 35144555 10.1186/s12879-022-07099-7 PMC8830018

